# Sentiments Regarding COVID-19 Vaccination among Graduate Students in Singapore

**DOI:** 10.3390/vaccines9101141

**Published:** 2021-10-06

**Authors:** Lee Jin Lim, Ashley J. W. Lim, Kevin K. Fong, Caroline G. Lee

**Affiliations:** 1 Department of Biochemistry, Yong Loo Lin School of Medicine, National University of Singapore, Singapore 117596, Singapore; bchllj@nus.edu.sg (L.J.L.); ash.ljw@nus.edu.sg (A.J.W.L.); 2 NUS Graduate School, National University of Singapore, Singapore 119077, Singapore; kevin.fong@u.nus.edu; 3 Cancer and Stem Cell Biology, Duke-NUS Medical School, Singapore 169857, Singapore; 4 National Cancer Centre Singapore, Division of Cellular & Molecular Research, Humphrey Oei Institute of Cancer Research, Singapore 169610, Singapore

**Keywords:** COVID-19, coronavirus, SARS-CoV-2, vaccination, vaccine acceptance, vaccine hesitancy, vaccine rejection, graduate student, Singapore

## Abstract

As the COVID-19 pandemic rages unabated, and with more infectious variants, vaccination may offer a way to transit out of strict restrictions on physical human interactions to curb the virus spread and prevent overwhelming the healthcare system. However, vaccine hesitancy threatens to significantly impact our progress towards achieving this. It is thus important to understand the sentiments regarding vaccination for different segments of the population to facilitate the development of effective strategies to persuade these groups. Here, we surveyed the COVID-19 vaccination sentiments among a highly educated group of graduate students from the National University of Singapore (NUS). Graduate students who are citizens of 54 different countries, mainly from Asia, pursue studies in diverse fields, with 32% expressing vaccine hesitancy. Citizenship, religion, country of undergraduate/postgraduate studies, exposure risk and field of study are significantly associated with vaccine sentiments. Students who are Chinese citizens or studied in Chinese Universities prior to joining NUS are more hesitant, while students of Indian descent or studied in India are less hesitant about vaccination. Side effects, safety issues and vaccine choice are the major concerns of the hesitant group. Hence, this study would facilitate the development of strategies that focus on these determinants to enhance vaccine acceptance.

## 1. Introduction

With >180 million confirmed cases and a death toll of >4 million worldwide as of July 2021 [1], the coronavirus disease 2019 (COVID-19) pandemic has thrown the world into an unprecedented cataclysmic global crisis that has not only taken a huge toll on our global health care system, but has also caused unparalleled economic and social disruption [2]. Countries scramble to contain the virus spread and “flatten the curve” to prevent total meltdown of their health care systems either by instituting full or partial lockdowns and strict border restrictions before an effective vaccine or treatment can be developed to lessen the impact of the virus’ devastation on human lives. Not only have these extreme measures on curtailing physical human interactions come at an enormous socioeconomic cost [3], they also have a significant, far-reaching impact on every aspect of our lives [4], including our mental health [5], as we pit life against human liberty [6].

The lightning-fast development of vaccines that was approved for emergency use as early as December 2020 [7] offered a glimmer of hope to bridle COVID-19 spread or at least mitigate serious illnesses and deaths, thus providing countries a pathway to ease strict restrictions on physical social interactions and travel borders to attain some normality [8,9]. However, vaccine hesitancy remains a major threat to mass vaccination efforts to immunize a sufficient proportion of the population to achieve herd immunity/protection [10,11,12,13]. Even before the COVID-19 pandemic, vaccine hesitancy has been highlighted by the World Health Organization (WHO) as one of the top threats to global health [14]. Coupled with heightened negative information and public suspicion about the speed of COVID-19 vaccine development [13], the considerable challenges posed by COVID-19 vaccine hesitancy is set to undermine any progress made to protect societies against COVID-19 unless more steps are taken to address these issues. It is thus imperative to understand the perceptions of different segments of the population towards COVID-19 vaccination to facilitate strategies to persuade the hesitant to be vaccinated not only for their own protection, but also for the protection of the community that they live in.

Given the voluntary nature of the COVID-19 vaccination exercise, its success in achieving herd immunity/protection would be largely dependent on the public’s acceptance and participation in the vaccination program. However, the speed of the COVID-19 vaccine development, the frequent updates, uncertainties over whether the vaccines are protective against newer COVID-19 variants, as well as the constantly emerging discoveries have led both healthcare professionals as well as the general public to employ a wait-and-see attitude towards vaccination, thus slowing vaccination efforts. A recent survey of sentiments regarding COVID-19 among Singaporean residents aged 21 years and above reported that only 67% of the respondents are willing to take the vaccine, 20% are unsure and 13% are unwilling to take the vaccine [15]. It is important to note that for a vaccine with 95% efficacy, herd immunity/protection can only be achieved with a vaccination rate of 63–76% [16,17]. The main concerns cited in the above-mentioned survey included vaccine safety and side effects (53%), as well as uncertainty about the vaccine efficacy (50%) [15]. In that same study, younger respondents (aged 21–39) are 1.25 times more likely to be concerned about vaccine safety compared to older (aged 60 and above) respondents [15].

Demographic factors that predicted vaccine acceptance among adults in Australia and healthcare workers in Asian countries were reported to include being female, above 55 years old, having a university degree, having children or dependents, and having more household income [18,19]. In a global and an Asia-Pacific study on healthcare workers, attitudinal correlates that predicted vaccine acceptance include confidence in government and healthcare systems, societal satisfaction, perceived severity of the pandemic, and perceived disruption due to the pandemic [19,20]. Behavioral correlates such as engaging in regular physical activity were also found to predict vaccine acceptance among the healthcare workers in Asian countries [19]. From a longitudinal study of Australians aged 18 years and above, concerns against vaccine include potential vaccine side effects and safety of the vaccine during development [21,22]

Here, we report the COVID-19 vaccination sentiments of graduate students studying at the National University of Singapore (NUS), the largest and most comprehensive University with the greatest number of graduate students in Singapore. Singapore is the 2nd most densely populated sovereign state in the world with a population of ~6 million living in a small island city-state of ~720 square kilometers [23], making it an ideal place for the rapid spread of any infectious agent if the country is ill-prepared. It is a multiracial and multicultural country with Chinese as a majority (74.2%), followed by Malays (13.2%) and then Indians (9.2%) [23], each with their own set of beliefs and cultural sensitivities. Thus far, with strict human restriction policies in place, Singapore has performed reasonably in curbing COVID-19 spread with ~68,000 confirmed cases and 55 deaths as of 1 September 2021 [24].

Singapore’s vaccination program with the Pfizer-BioNTech vaccine started on 30 December 2020, on a free and voluntary basis for all Singaporeans and long-term residents [25]. It is being progressively rolled out to different groups based on their risk levels, with priority given to front-line health workers and elderly persons aged 70 and above. Vaccination is conducted through 38 vaccination centers located across the island, with more opening up progressively [26].

Graduate students represent the segment of population that are the most highly educated and hence are perceived to be less biased and more responsive to public health issues [27,28]. As Singapore is a society that values education, the views of highly educated individuals can be influential. Hence, it is important to better understand their sentiments about COVID-19 vaccination of this group of the population as they have potential to be ambassadors to help educate the public about the benefits of vaccination.

It is thus timely to examine COVID-19 vaccination sentiments of graduate students at NUS which represent a highly educated but diverse population, being citizens of >50 countries and were educated in nearly as many countries prior to pursuing their graduate studies at NUS, although a majority of these students are Asian and studied in Asia. This diversity provides us with an opportunity to explore whether citizenship or prior education background influence their perceptions of COVID-19 vaccination. Furthermore, as a comprehensive University with diverse fields of study at the graduate level, it is also worthwhile to examine if field of study that these students pursue affect their COVID-19 vaccination sentiments as this factor was previously reported to influence their perception of COVID-19 vaccination with those in the sciences or health-related course being more positive about vaccination [19,29,30].

## 2. Methods

### 2.1. Materials and Procedure

The COVID-19 vaccination data was based on archival data collected by the NUS Graduate School (NUS GS) to identify risk level and to evaluate if graduate students should be prioritized for vaccination. An online survey link was sent to all PhD and Masters by Research graduate students at NUS through their student email and the survey was made available from 19 February 2021 to 28 February 2021. The study was approved by the Institutional Review Board of NUS Institutional Review Board (NUS-IRB), Singapore (Approval: NUS-IRB-2021-383, Date: 21 May 2021).

The survey assessed the following:

(1) COVID-19 infection status (Yes/No);
(2) COVID-19 vaccination status (Complete/ Incomplete/ Scheduled/No);
(3) COVID-19 Risk (Thesis research requires them to work in an environment that puts them at high-risk for COVID-19 infection)
(4) Contraindication to COVID-19 vaccine (Pregnancy/Immunocompromised/Previous anaphylaxis or severe allergy reaction/ Breastfeeding/ Bleeding disorder/None of the above);
(5) Willingness to be vaccinated (If you are given an opportunity to be vaccinated, will you choose to be vaccinated? Yes/No/Unsure);
(6) Concerns for the COVID-19 vaccination (Response can cite multiple concerns):
  - Safety Concerns
  - Side Effects
  - Vaccine Choice
  - Unnecessary
  - Youth (I’m young and healthy)
  - Personal (allergy/pregnancy/Others). Only respondents who responded No or Unsure answered this question.

Other demographic data, such as age, gender, nationality, and undergraduate university records, were gathered from their student’s record and summarized in Table S1.

### 2.2. Statistical Analysis

For statistical analysis, vaccination sentiments were divided into two categories, which were “Willing” and “Hesitant”. The latter was a combination of “No” and “Unsure” response to the “Willingness to be vaccinated” question in the survey. Association between independent variables with vaccine acceptance were assessed using the Chi-Square test. To evaluate for significant association between levels within each independent variable (e.g., religion, citizenship, etc.) with vaccination sentiments, a univariate logistic regression was used. A multivariate Rpart (Recursive Partitioning and Regression Trees) (4.1–15) [31,32] was used to identify potential predictors of vaccine acceptance among the independent variables. Bonferroni adjusted *p*-value of less than 0.05 was considered as statistically significant in relevant statistical analyses and the analyses was performed using R version 4.0.3 [33].

## 3. Results

### 3.1. Majority of Respondents Are Asian PhD Graduate Students Pursuing STEM Fields

**Figure 1:**
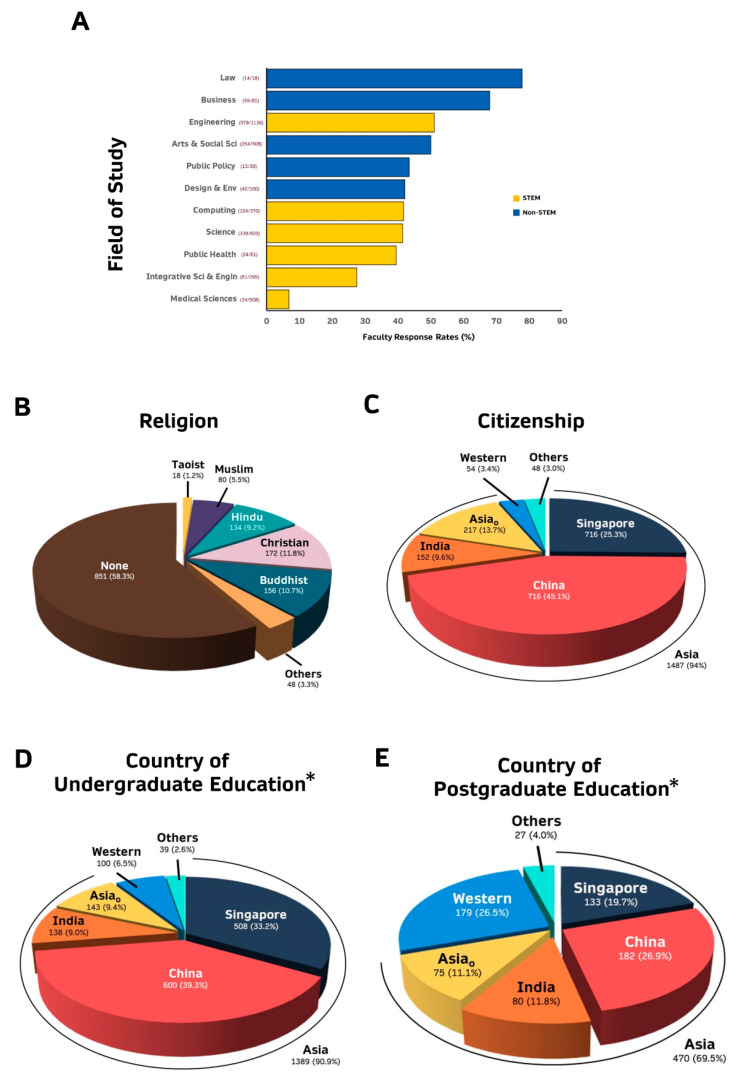
Proportion of respondents based on: (**A**) Field of Study (Medical Sciences: include those in the Faculties of Medicine, Dentistry and Duke-NUS Medical School); (**B**) Religion; (**C**) Citizenship (See Table S2 for the specific countries for Asia_o_, Western and Others); (**D**) Country of Undergraduate Education * (See Table S2 for the specific countries for Asia_o_, Western and Others) and (**E**) Country of Postgraduate Education * (See Table S2 for the specific countries for Asia_o_, Western and Others). * For students who studied in universities from more than one country, all the different countries are included.

Approximately 42.8% (1691/3950) of our graduate students completed the survey. 101 respondents were excluded from further analysis as their records were not found in our system, suggesting that they are not bona vide graduate students from NUS. The remaining 1589 respondents represent 40.22% of our PhD and Masters by Research graduate student population. A majority of the respondents are pursuing PhD (87.5%) and in the STEM (Science, Technology, Engineering and Math) (76.2%) fields (Table S1). Highest response was received from graduate students pursuing Law and Business and lowest response from students pursuing Medical Sciences (Duke-NUS, Dentistry and Medicine) (Figure 1A). More of the respondents were males (*n* = 935, 58.84%) and have a mean age of 27.66 years old (SD = 4.57).

A majority of these graduate students have no religion (>58%), while ~9–12% are Hindus, Buddhists, or Christians (Figure 1B). Respondents are citizens of 54 countries (Table S2), mostly of Asian origin (94%) with the majority being citizens of China (45.1%), Singapore (25.3%) and India (9.6%) (Figure 1C). Prior to joining the graduate programs at NUS, these respondents did their undergraduate (UG) studies at universities in 50 countries (Table S2). The percentage of respondents who pursued their undergraduate (UG) degree in China (39.3%) (Figure 1D) is lower than the percentage of Chinese citizens (45.1%) (Figure 1C), while those who pursued their UG education in Singapore (33.2%) and Western (6.5%) countries (Figure 1D) is higher than percentage of Singapore (25.3%) or Western (3.4%) citizens (Figure 1C). Approximately 43% of these NUS graduate respondents also pursued postgraduate (PG) education in 44 different countries (Table S2) prior to joining NUS, with a greater proportion doing their PG education in Western (26.5%) countries and less in China (26.9%) compared to their UG education (Figure 1E).

### 3.2. Non-Religious, Citizens of China and Graduate Students Pursuing Engineering, Law, Business and Design and Environment Are Less Willing to Be Vaccinated

**Figure 2:**
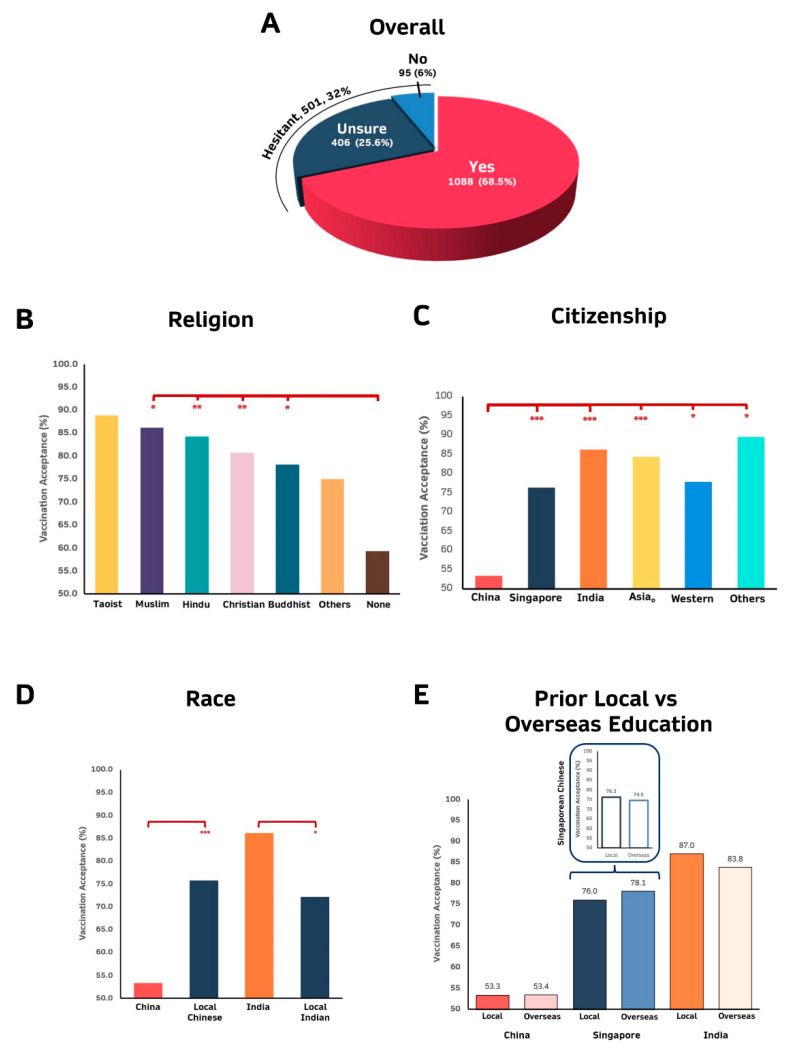

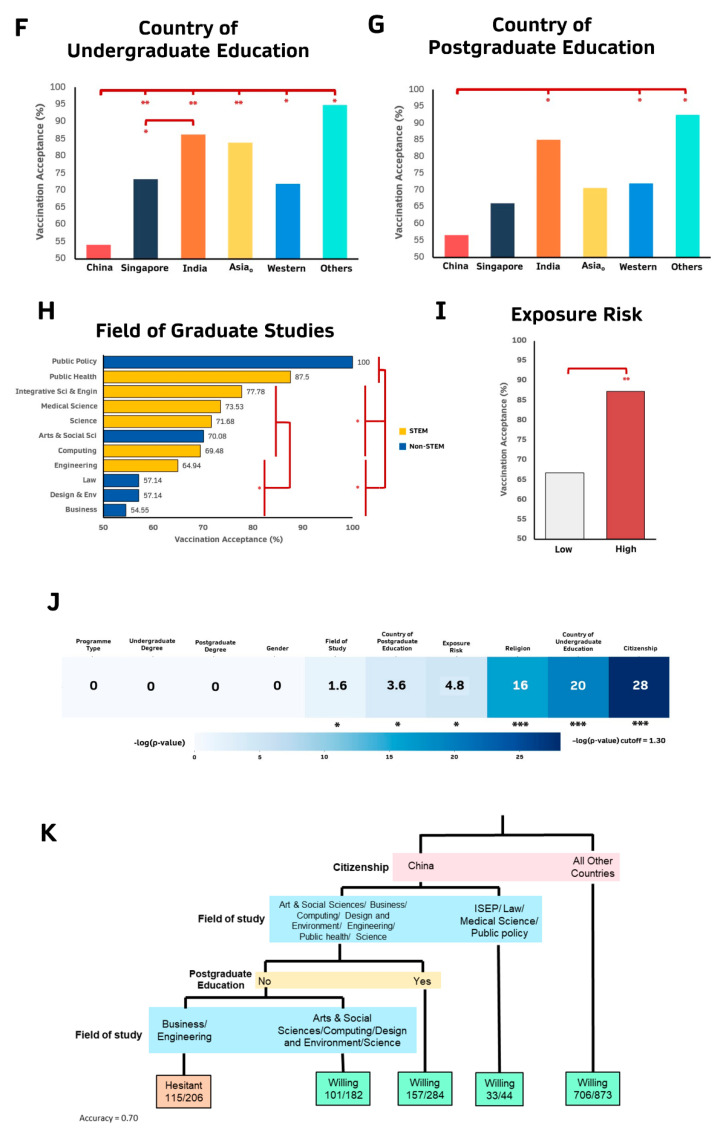
Percentage of respondents who are willing to be vaccinated. (**A**) Overall. (**B**) Religion; (**C**) Citizenship (See Table S2 for the specific countries for Asia_o_, Western and Others); (**D**) Race; (**E**) Prior Local or Overseas Education. (**F**) Country of Undergraduate Education (See Table S2 for the specific countries for Asia_o_, Western and Others). (**G**) Country of Postgraduate Education (See Table S2 for the specific countries for Asia_o_, Western and Others). (**H**) Field of Graduate Studies and (**I**) Risk Exposure of Thesis Project. (**J**) Chi-Square association of the different independent variables. (**K**) Predictors of Vaccine Hesitancy using Recursive Partitioning and Regression Trees (Accuracy = 0.7). * adjusted *p* < 0.05, ** adjusted *p* < 10^−5^ and *** adjusted *p* < 10^−10^. Details of the statistics including OR and CI are found in the Supplementary Figure S1.

A majority (68.5%) of the respondents were willing to be vaccinated with COVID-19 vaccines while 32% were hesitant. These hesitant respondents comprise 25.6% unsure and 6% unwilling respondents (Figure 2A). Neither gender nor level (PhD versus Masters) of graduate studies pursued were significantly associated with vaccine sentiments (adjusted *p*-value = 1). Non-religious graduate students are least willing to be vaccinated compared to those who declared at least a religion with >80% of Taoist, Muslims, Hindus and Christians expressing willingness to be vaccinated (odds ratios (OR): 2.392 to 4.305, adjusted *p* < 0.05) (Figure 2B and Figure S1A).

NUS graduate students who are citizens of China are the least willing to be vaccinated compared to citizens of other countries (OR: 2.825 to 7.519; adjusted *p* < 0.05). (Figure 2C and Figure S1B). As Singapore is a multi-racial society comprising ~74% Chinese and ~9% Indians [34], we evaluated COVID-19 vaccination sentiments of Singapore Chinese and Singapore Indians compared to China Chinese and India Indians. As shown in Figure 2D (and Figure S1C), compared to China Chinese, Singaporean Chinese are more willing to be vaccinated (OR = 2.74, Adjusted *p* = 7.723 × 10^−12^), while Singaporean Indians are less willing to be vaccinated compared to India Indians (OR = 0.416, Adjusted *p* = 4.676 × 10^−2^). We then investigated whether exposure to overseas education would influence sentiments towards COVID-19 vaccination of the 3 major citizen groups of our graduate students, namely Chinese, Singaporeans and Indians. As evident in Figure 2E, exposure to overseas education did not significantly alter the sentiments of the respective citizens although there is a slightly more positive sentiments (76 to 78%) towards vaccination of Singaporeans who were educated overseas and slightly more negative sentiments towards vaccination amongst Singaporean Chinese (76.3 to 74.5%) and Indian nationals (87 to 83%) who studied overseas. Consistent with trends observed for citizenship, NUS graduate students who previously pursued their undergraduate and/or postgraduate studies in China were the most hesitant to be vaccinated (Figure 2F,G, Figure S1D,E).

Unsurprisingly, graduate students in fields that influence public policies (Public Policy and Public Health) are the most willing, while in contrast, those in practice professions including engineering, law, business and design and environment are the most reluctant about vaccination (ORs = 0.153, Adjusted *p* = 6.04 × 10^−3^) (Figure 2H and Figure S1G). Similarly, graduate students whose thesis project requires them to work in environment with higher risk for COVID-19 infection (e.g., hospitals) are more willing to be vaccinated compared to those not working in high-risk environments (OR = 3.43, Adjusted *p* = 3.421 × 10^−6^) (Figure 2I and Figure S1F).

### 3.3. Chinese Citizens without Prior Postgraduate Education Pursuing Business/Engineering Fields Are Predicted to Be Hesitant to Be Vaccinated

In summary, of the different variables examined, six factors were found to be significantly associated with vaccine sentiments. Citizenship is the most significantly associated followed by country of undergraduate education, religion, exposure risk, country of postgraduate education and finally, field of study (adj *p* > 0.05) (Figure 2J). The multivariate Recursive Partitioning and Regression Trees was then performed to predict the characteristics of postgraduate students who are most likely to be hesitant to be vaccinated while accounting for possible confounding effects [35]. As evident from Figure 2K, Chinese citizens without prior postgraduate education and pursuing either Business or Engineering field of studies are predicted to be hesitant about COVID-19 vaccination (Accuracy= 0.70). This observation is consistent with the above observation that these 3 factors are significantly associated with vaccine sentiments (Figure 2J).

### 3.4. Safety Issues, Side Effects and Vaccine Choice Are the 3 Most Common Reasons for Vaccination Hesitancy

**Figure 3:**
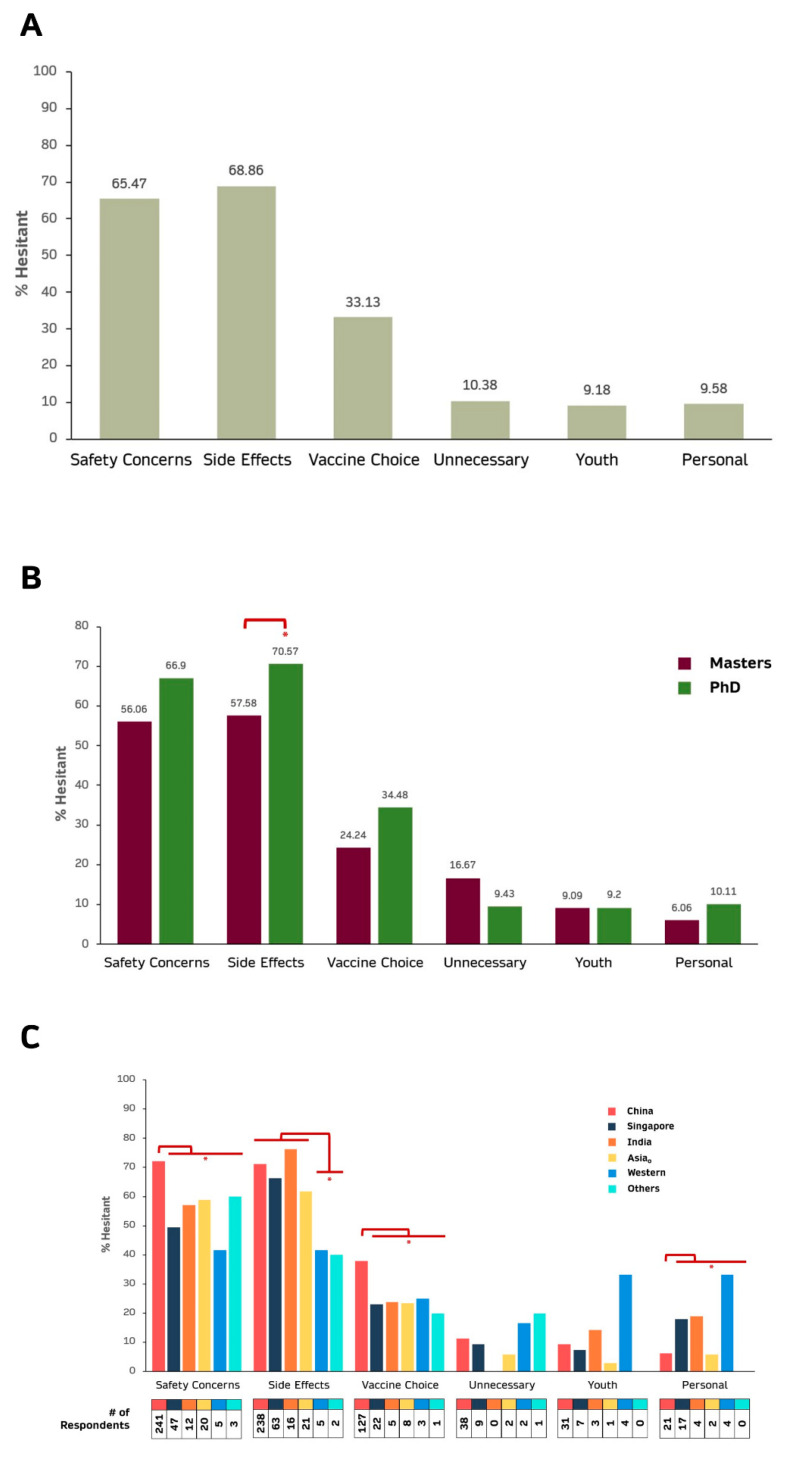
Reasons for being hesitant about COVID-19 vaccination among hesitant respondents. (**A**) Overall. (**B**) Programme types; (**C**) Citizenship (See Table S2 for the specific countries for Asia_o_, Western and Others). * adjusted *p* < 0.05.

Vaccine safety issues and side effects were the 2 most common concerns amongst the ~30% vaccine hesitant graduate students while vaccine choice is the 3rd most common reason (Figure 3A). Generally, a larger proportion of PhD compared to Masters students felt that Safety, Side Effects, Vaccine Choice and Personal Reasons (pregnancy, immunocompromise, severe allergy, breast-feeding, bleeding disorders, etc.) were reasons for their hesitancy with only Side Effects showing statistical significance (OR = 1.767, Adj *p* = 3.519 × 10^−2^) (Figure 3B and Figure S2A). On the other hand, more Masters compared to PhD students felt that vaccines were unnecessary (Figure 3B).

Citizens of different countries generally also highlighted the same 2 concerns (safety, side effect) as the main reasons for their hesitancy (Figure 3C). Postgraduate students who are of Western citizenship do not seem to have any dominant concerns with greater percentage expressing personal reasons or being young as reasons for their hesitancy compared to citizens of other country (Figure 3C). A majority (63%) of postgraduate students expressing hesitancy towards COVID-19 vaccination are of Chinese citizenship. Compared to Chinese citizens, significantly lower percentage of postgraduate students of other citizenship highlighted safety (OR = 0.419, *p* = 1.069 × 10^−5^) and vaccine choice (OR = 0.496, *p* = 1.123 × 10^−3^) as reasons for their hesitancy. Conversely, a significantly greater percentage of other citizenship expressed personal issues (OR = 2.874, *p* = 6.129 × 10^−4^) as important concerns (Figure 3C and Figure S2B). Interestingly, a significantly lower percentage of students from non-Asian countries expressed side effects as a reason for hesitancy compared to postgraduate students from Asian countries (OR = 0.302, *p* = 1.733 × 10^−2^).

## 4. Discussion

The survey in this cross-sectional study was performed in mid to the end of February 2021, when the Pfizer-BioNTech COVID-19 vaccine was available in Singapore and high-risk individuals, e.g., frontline healthcare workers were prioritized to be vaccinated. It was conducted primarily to identify NUS graduate students, whose thesis research project puts them at higher risk of COVID-19 infection (e.g., working with human samples or in a high-risk environment or interacting with individuals at high-risk of contracting COVID-19). This is to facilitate an appeal to the Singapore Ministry of Health to provide these students with earlier access to COVID-19 vaccination to minimize disruption to their graduate education. At the juncture of the survey, a majority of graduate students pursuing medical sciences at the Yong Loo Lin School of Medicine, Dentistry and Duke-NUS Medical School who chose to be vaccinated would have received at least one dose of the vaccine, which likely accounted for the very low response (<10%) from these fields of studies compared to the other non-medical sciences faculties (Figure 1A).

Amongst the graduate students who responded, a similar percentage of these students (~68.5%) (Figure 2A) were willing to be vaccinated compared to the general Singapore population around the same period (February to March 2021: 60–70%) [15,36]. However, less NUS graduate students were unwilling (~6%) while more were unsure (25.6%) (Figure 2A) compared to the general population (unwilling: 13–20%; unsure: 20–22%) [15]. This is consistent with the notion that while highly educated individuals can better appreciate the value of vaccination, they require additional evidence before fully embracing it. It is also consistent with the observation that the major reasons for hesitancy (unsure or no) were vaccine safety, side effects and vaccine choice (Figure 3A).

NUS postgraduate students seemed less willing to be vaccinated (~68.5%) compared to University students from other countries, including college students from China (76.3%) [27], medical students in the USA (77%) [37], undergraduate medical students in India (89.4%) [38], University students in Canada (77.8 to 79.6%) [39], as well as University students in Italy (86.1%) [28]. Consistent with previous reports of high COVID-19 vaccine acceptance (89.4%) [38] by University students in India, NUS graduate students who are citizens of India or had their undergraduate/postgraduate education in India are also very willing to be vaccinated (≥85%) (Figure 2C,F,G). Curiously, on the other hand, while it was previously reported that college students in China are reasonably willing to be vaccinated (76.3%) when they were surveyed between 27 December 2020 to 18 January 2021 [27], current NUS postgraduate students who are Chinese citizens or pursued their undergraduate/postgraduate studies in China were more hesitant (<57%) about COVID-19 vaccination when they were surveyed between 19 February 2021 to 28 February 2021 (Figure 2C,F,G). This is regardless of whether these graduate students of Chinese citizenship were exposed to overseas education at least once in their life (Figure 2E). However, Singaporeans living in a multi-racial society who are ethnically Chinese were significantly more willing to be vaccinated than the Chinese in China (OR = 2.74, Adj *p* = 7.723 × 10^−12^) (Figure 2D), suggesting that educational exposure alone may be insufficient and a longer-term integration into a different culture or being part of that culture since birth may be required to modulate COVID-19 vaccine sentiments. Interestingly, majority of the Chinese citizens declared that they do not have any religion (94.8%) (data not shown) and NUS graduate students with no religion were found to be the least willing to be vaccinated (58.7%) (Figure 2B and Figure S3). This is consistent with a recent report that contrary to the belief that the evangelical Christians in the USA were the most hesitant to receive COVID-19 vaccination, it was the secular segment of the population without any religious affiliations, who were the least likely to have been vaccinated with any COVID-19 vaccine [40]. This is also consistent with the observation that lower (although not significant) percentage of postgraduate students who are Chinese citizens without any religion was willing to be vaccinated (Figure S3) compared to those who has religion. However, similar trends were not observed for Singapore citizens as there are very little difference between those with religion and those without (Figure S3). It would thus be worthwhile to further explore whether secularism or citizenship or an interaction between the two play a more important role in modulating COVID-19 vaccine sentiments to facilitate the design of strategies targeting the appropriate group.

The discrepancy between the percentage of university students in China (76%) [27] and NUS postgraduate students who are Chinese citizens (<57%) (Figure 2C) willing to be vaccinated could be due to a few factors. With the rapidly evolving deluge of information about the COVID-19 virus, its variants and its vaccines, the period at which the survey was conducted may influence sentiments about COVID-19 vaccination. To date (1 September 2021), nearly 80% of the general population [41,42] and >85% of the population at the same age range (12–39 years old) as our graduate students [43] have been fully vaccinated in Singapore, suggesting that many of those who were surveyed to be hesitant in our survey in February 2021 may have now been vaccinated as their sentiments may have changed with additional information. A longitudinal study would be useful to evaluate changes in sentiments over time. Differences in the survey method may be another reason for the discrepancy. While the previous study performed snowball sampling, in this study, the survey was sent via email from the NUS Graduate School to all postgraduate students for their voluntary participation. Snowball sampling used in the previous study is dependent on the social networks of participants and may be prone to sampling bias leading to recruitment of participants with similar thinking and sentiments towards specific issues, thus making it difficult to generalize beyond the sample studied [44,45,46]. The age of participants of the study may also account for differences in their sentiments. While the previous study surveyed were mainly undergraduate students between the age of 16 and 30 (mean age: 19–20), our study surveyed postgraduate students of Chinese citizenship who are between age of 18–39 (mean age: 25–26). However, in our study, the younger respondents were only slightly more vaccine hesitant (i.e. unsure or unwilling) compared to older respondents (OR = 1.04; 95% CI (1.02–1.07), *p* < 0.001) (data not shown), which is consistent with some studies reporting that the young are more likely to be hesitant about receiving COVID-19 vaccination [40,47,48]. Hence, contribution of age to the discrepancy remains unclear. As disease-related perception (including perceived susceptibility to infection and perceived severity of the disease when infected) [49] may influence one’s willingness to be vaccinated, another likely explanation for the discrepancy could be differences in the disease-related perception of students residing in China or Singapore. China was the first country to experience the COVID-19 pandemic and the extent this disease can severely sicken the infected, overwhelming the healthcare system and causing death. Majority (~90%) of the population of China were reported to be willing to be vaccinated with COVID-19 vaccines [20]. Hence, students residing in China are more likely to better appreciate the likelihood of getting infected with the COVID-19 virus as well as appreciate the severity of the disease once they are infected. On the other hand, there were very few community cases (≤2 per day) in Singapore at the point when this survey was conducted [24,50]. Although the total cumulative number of individuals infected with COVID-19 in Singapore was quite high (~60,000 by 28 February 2021), it primarily affected only a single isolated segment of the population living in dormitories (~54,511) or imported (~3100) with very low community spread [24]. As those infected in Singapore then were young and strong, the number of deaths from COVID-19 infection was low. Hence, students residing in Singapore may feel less urgency to be vaccinated than students residing in China. This would account for more ‘unsure’ but less ‘no’ to vaccination in our survey. With the new, more infectious delta variant raging in the world recently that was reported to also affect the younger population [51,52], it would be worthwhile to evaluate whether the sentiments of these same students in Singapore towards vaccination have changed with the current fluctuating community spread in Singapore.

This study also highlights that the field of study that these postgraduate students pursue influence their sentiments towards COVID-19 vaccination. Postgraduate students studying to be policy makers (Public Policy or Public Health) are the most willing to be vaccinated, while those in more practical fields like Business and Law are the least willing (Figure 2H). The others in the more academic fields of studies were also reasonably willing to be vaccinated (>70%) (Figure 2H).

Overall, citizenship, country of undergraduate and postgraduate education, religion, exposure risk and field of studies were found to be significant factors modulating COVID-19 vaccination sentiments (Figure 2J). Notably, graduate students who are Chinese citizens with no prior postgraduate education and pursuing practical fields of studies, namely, Business or Engineering are predicted to be hesitant about COVID-19 vaccination based on our multivariate Rpart analysis. Hence, specific public health campaigns can be targeted towards these students.

The main concerns among the hesitant group are safety, side effects and vaccine choice (Figure 3A), which are also the concerns highlighted in other studies [21,53,54,55,56,57,58]. Significantly higher proportion of hesitant PhD students are concerned about the side effects of the COVID-19 compared to hesitant Masters’ students (Figure 3B and Figure S2A). Compared to citizens of other countries, a significantly greater percentage of Chinese postgraduate students highlighted safety and vaccine choice as a reason for their hesitancy (Figure 3C and Figure S2B). Conversely, significantly lower percentage of Chinese postgraduate students highlighted personal issues as a reason for their hesitancy. While the exact sentiments about vaccine choice remain unclear, amongst the few optional free-text comments that were received were remarks from students who expressed preference for vaccines made in China (data not shown). At the point of data collection, only the USA/Germany-made Pfizer vaccine were available in Singapore, while the USA-manufactured Moderna vaccine was deployed after the survey (17 March 2021) [59,60]. Country of origin of vaccine was also previously reported to be an important factor. Respondents from Israel preferred vaccines manufactured in USA/UK rather than China [61], while respondents from China expressed preference for China-made over foreign-made COVID-19 vaccines [62]. With the world preparing to ease travel restrictions when the pandemic subsides, different countries have imposed different travel restrictions that only allow those vaccinated with specific vaccines developed in specific countries through their borders [63]. In fact, only those who are vaccinated with China-made vaccines will find it easier to enter China [63,64,65,66] which may perhaps explain why NUS postgraduate students who are Chinese citizens are more concerned about the type of vaccines they receive. With Singapore now recognizing all COVID-19 vaccines, including China-made vaccines, that are approved under the Emergency Use Listing (EUL) by the World Health Organization (WHO) as of 10 August 2021 [67], it is worthwhile to re-evaluate the COVID-19 vaccine sentiments of these students as there are now greater choice of different vaccines in Singapore. Hence, addressing these major concerns in the specific target groups are key to encouraging widespread acceptance of the COVID-19 vaccine.

One major limitation is that this is a cross-sectional study in which data was collected at only a single time point, with availability of only the USA/Germany-made Pfizer vaccine and before the vaccines were readily available to all residents in Singapore including graduate students. Hence, this study can only probe intention/sentiments, but not the actual behavior towards COVID vaccination amongst the graduate students. As information about the COVID-19 virus and its variants, as well as the diverse vaccines available, is rapidly evolving, sentiments with regards to COVID-19 vaccination are thus likely to change. In fact, the percentage of the Singapore population willing to be vaccinated has increased from 60–70% in February/March 2021 to >90% in July 2021 [36]. Furthermore, to date (1 September 2021), nearly 80% of the general population [41,42] and >85% of the population of similar age (12–39 years old) as our graduate students [41] in Singapore have been fully vaccinated suggesting that many of those who were hesitant at the point of the survey would have been vaccinated. Hence, a longitudinal study will facilitate deeper insights into critical factors that drive change in sentiments towards COVID-19 vaccination. Another limitation is the greatly uneven distribution of respondents who are citizens of the different countries as well as the different fields of studies limiting statistical comparisons amongst some combinations. Postgraduate students pursuing Medical Sciences in Singapore were prioritized to be or have already been vaccinated at the point of the survey, leading to very low response from this group of students to the survey (Figure 1A), hence possibly skewing the results as these students are likely to be amongst the group of postgraduate students who are most willing to be vaccinated as evident from previous studies [19,37,38,68], as well as Figure 2H. Another potential limitation is that this study only examined postgraduate students from a single University which may not represent all postgraduate students in Singapore. Nonetheless, NUS, which is the focus of this study is the largest and most comprehensive University with the greatest number of graduate students in Singapore. Another potential limitation is that this study only superficially examined six common concerns among the hesitant group. Future studies could probe deeper into issues leading to COVID-19 vaccine hesitancy including comprehensively examining the attitudinal and behavioral factors and specific vaccine apprehension, as well as fully investigating the 5C drivers of vaccine hesitancy including Confidence, Complacency, Convenience/Constraints), risk Calculation and Collective responsibility [53,59]. Future studies could also explore different approaches to survey sentiments, including social media sentiment analyses [69] and COVID-19 community temporal analyzer [70] to shed additional light on the COVID-19 vaccine sentiments.

In conclusion, while a majority of graduate students at NUS at the point of the survey were willing to be vaccinated, approximately a quarter (25.6%) were unsure, perhaps reflecting the cautiousness of graduate students, awaiting more evidence before deciding. Notably, postgraduate students who are Chinese citizens, without prior postgraduate education, pursuing practical fields of studies (engineering and business) were predicted to be hesitant about COVID-19 vaccination. Safety, side effect and vaccine choice are major concerns among the hesitant postgraduate students. Hence, findings from this study can facilitate the development of strategies to focus on the specific concerns as well as the specific target group of postgraduate students, whose views are highly regarded especially in Asian societies, as they are potential ambassadors who can influence public vaccination sentiments.

## Data Availability

The analyzed data that were used is available from the author on reasonable request.

## Supplementary Materials

The following are available online at https://www.mdpi.com/article/10.3390/vaccines9101141/s1, Figure S1: Odd ratio plots for demographic variables of the graduate students, Figure S2: Odd ratio plots for vaccination concerns, Figure S3: Religion and vaccination sentiments, Table S1: Summary for the background of the graduate students who responded to the survey, Table S2: Specific countries for citizenship, undergraduate education and postgraduate education of graduate students who responded to the survey.
