# Supplementary material for: Sentiments Regarding COVID-19 Vaccination among Graduate Students in Singapore
Source: Vaccines (Basel). 2021 Oct 6;9(10):1141. doi: 10.3390/vaccines9101141 (PMC8538718; doi:10.3390/vaccines9101141)
Supplement: Supplementary file 1 [file vaccines-09-01141-s001.zip › vaccines-1386255-supplementary.pdf]

Supplemental Information

Sentiments regarding COVID-19 Vaccination among Graduate Students in Singapore

Lee Jin Lim1†, Ashley J.W. Lim1†, Kevin K. Fong2† and Caroline G. Lee 1,2,3,4*

*Corresponding authors' email: bchleec@nus.edu.sg

This file includes:

Figure S1 2
Figure S2 3
Figure S3 4
Table S1 5
Table S2 6

Figure S1

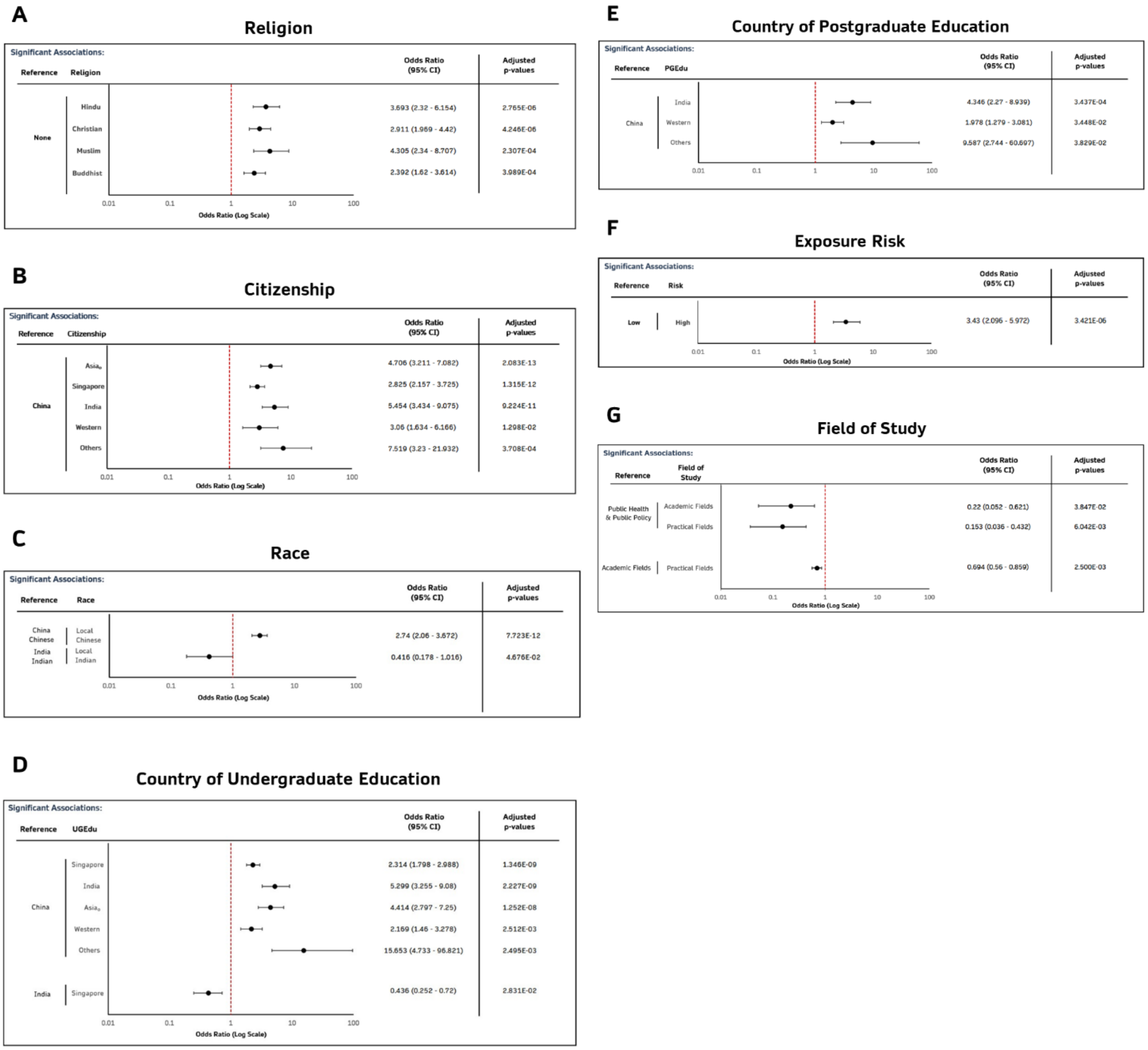

Figure S1. Odd ratio plots for demographic variables of the graduate students. The plots only show the associations that are significant after Bonferroni correction (Adjusted *P*-value <0.05).

Figure S2

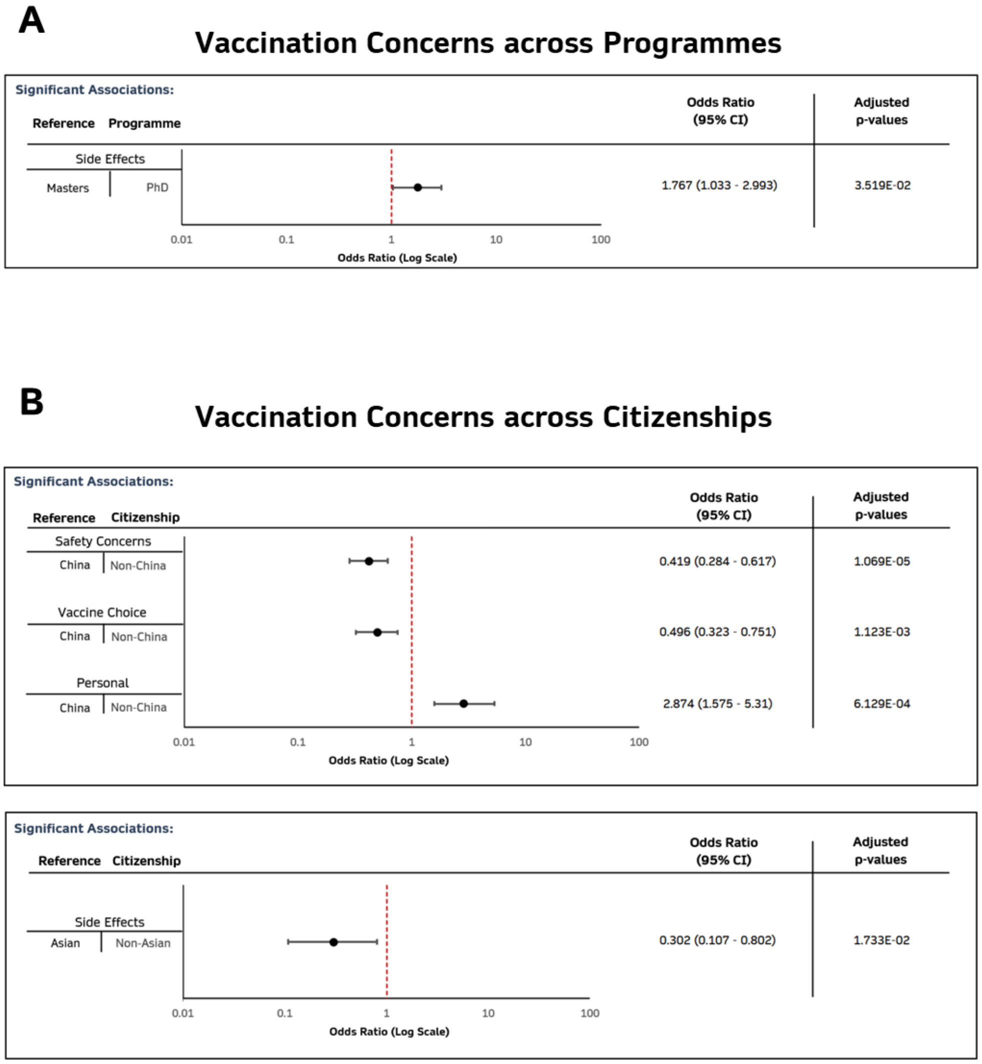

Figure S2. Odd ratio plots for vaccination concerns. The plots only show the associations that are significant after Bonferroni correction (Adjusted *P*-value <0.05).

Figure S3

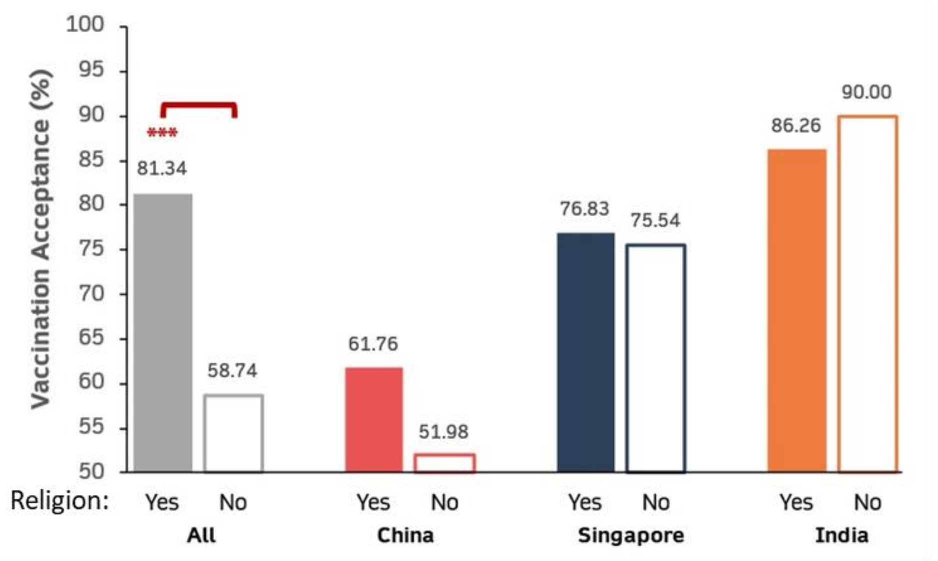

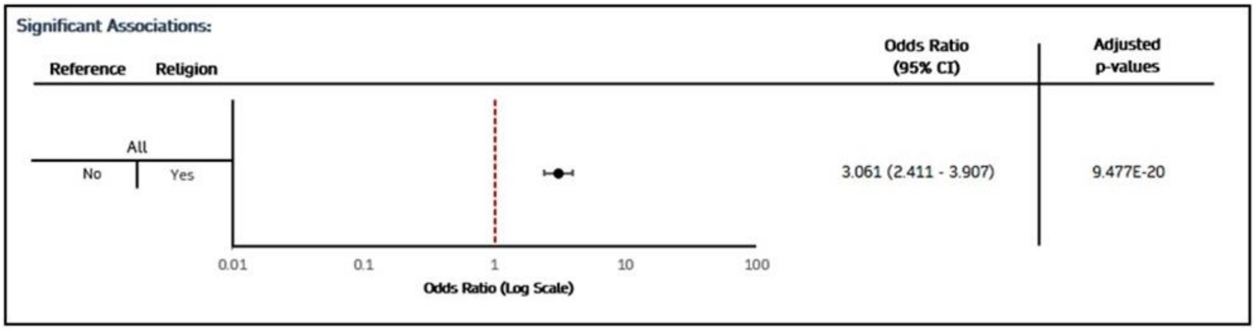

Figure S3. Religion and vaccination sentiments. (Top) Bar chart shows the percentage of vaccination acceptance of religious and non-religious graduate students from all countries, China, Singapore, and India respectively. (Bottom) Odd ratio plots for religion and vaccination sentiments. The plot only shows the association that is significant after Bonferroni correction (Adjusted P-value <0.05).

Table S1. Summary for the background of the graduate students who responded to the survey.

	Variables		Number (Percentage)
Demographic	Age (mean (SD))		27.60 (4.55)
	Gender (%)	Female	654 (41.2)
		Male	935 (58.8)
	Race (%)	Chinese	339 (85.8)
		Indian	37 (9.4)
		Malay	14 (3.5)
		Others	5 (1.3)
	Religion (%)	Buddhist	157 (10.7)
		Christian	173 (11.8)
		Hindu	134 (9.2)
		Muslim	80 (5.5)
		Taoist	18 (1.2)
		Others	48 (3.3)
		None	854 (58.3)
	Citizenship group (%)	Asia	217 (13.7)
		China	716 (45.1)
		Singapore	402 (25.3)
		Western	69 (4.3)
		India	152 (9.6)
		Others	33 (2.1)
Education	Programme type (%)	Master	199 (12.5)
		PhD	1390 (87.5)
	Field of study (%)	Arts & Social Sciences	254 (16.0)
		Business	55 (3.5)
		Computing	154 (9.7)
		Design and Environment	42 (2.6)
		Engineering	579 (36.4)
		ISEP	81 (5.1)
		Law	14 (0.9)
		Medical science	34 (2.1)
		Public Health	24 (1.5)
		Public Policy	13 (0.8)
		Science	339 (21.3)
	STEM (%)	Non-STEM	378 (23.8)
		STEM	1211 (76.2)
Others	Exposure risk (%)	No	1455 (91.6)
		Yes	134 (8.4)
	Vaccination (%)	No	501 (31.5)
		Yes	1088 (68.5)

SD: Standard deviation; ISEP: Integrative Science and Engineering;
STEM: Science, Technology, Engineering and Math

Table S2. Specific countries for citizenship, undergraduate education and postgraduate education of graduate students who responded to the survey.

No.	Country			Citizenship	Undergraduate	Postgraduate
1	Asia		Singapore			
2			China			
3			India			
4		$Asia_o$ (Others)	Malaysia			
5			Indonesia			
6			Korea, Republic of			
7			Thailand			
8			Sri Lanka			
9			Philippines			
10			Taiwan			
11			Hong Kong SAR, China			
12			Bangladesh			
13			Pakistan			
14			Japan			
15			Nepal			
16			Vietnam			
17			Cambodia			
18			Myanmar			
19			Lao People's Democratic Rep			
20			Macao SAR, China			
21	Western		United States			
22			United Kingdom			
23			Netherlands			
24			France			
25			Canada			
26			Germany			
27			Italy			
28			Peru			
29			Brazil			
30			Spain			
31			Hungary			
32			Finland			
33			Mexico			
34			Poland			
35			Switzerland			
36			Malta			
37			New Zealand			
38			Colombia			
39			Denmark			
40			Britain			
41			Australia			
42			Norway			
43			Sweden			
44			Costa Rica			
45			Ireland			
46	Others		Turkey			
47			Iran (Islamic Republic Of)			
48			Russian Federation			
49			Egypt			
50			Romania			
51			Israel			
52			Republic of Serbia			
53			Morocco			
54			Kazakhstan			
55			Greece			
56			Palestinian Territory, Occupied			
57			Uzbekistan			
58			Ghana			
59			Mauritius			
60			Saudi Arabia			
61			Lebanon			

Countries in "Asia" refer to countries in Southern, Eastern and South-Eastern Asia (https://www.worldometers.info/geography/how-many-countries-in-asia/). Countries in "Western" are the western countries that are defined in https://worldpopulationreview.com/country-rankings/western-countries. Countries that are not under "Asia" and "Western" categories are labelled as "Others". Grey box: Not present in this survey for the specific category.
